# Application of different laboratory techniques to monitor the behaviour of a *Mycoplasma synoviae* vaccine (MS-H) in broiler breeders

**DOI:** 10.1186/s12917-018-1669-8

**Published:** 2018-11-20

**Authors:** M. L. Moronato, M. Cecchinato, G. Facchetti, M. Mainenti, F. Gobbo, S. Catania

**Affiliations:** 1 Department of Animal Medicine Production and Health - MAPS, Università degli Studi di Padova, Viale dell’Università 16, 35020. Legnaro, Padova, Italy; 2 Avian Medicine Laboratory Mycoplasma Unit - SCT1, Istituto Zooprofilattico Sperimentale delle Venezie, Viale dell’Università 10, 35020. Legnaro, Padova, Italy; 3 Veterinary practitioner. Avicola Alimentare Monteverde SRL. Via San Donato, 107, 25038. Rovato, Brescia, Italy; 4 Istituto Zooprofilattico Sperimentale di Verona, SCT1, Via S. Giacomo 5, 37135 Verona, Italy

**Keywords:** *Mycoplasma synoviae*, MS-H, Chicken breeder, Monitoring, Genotyping

## Abstract

**Background:**

*Mycoplasma synoviae* (MS) is a major poultry pathogen which causes severe economic losses in all the productive sectors. The prevalence of MS in European countries has increased in the last few years, leading to greater attention to the available methods to prevent its spread. The main strategy currently applied for its containment is the development and maintenance of MS-free breeder flocks. A live MS vaccine (MS-H) obtained by mutagenizing an Australian field strain has recently been introduced in Italy. The aim of the present study was to evaluate the vaccine behaviour in broiler breeder groups at different production stages and the effectiveness of the available laboratory tests in discriminating the MS-H from a field strain.

**Results:**

The vaccine diffused extensively through the population, shown by the wide serological response (over 80% of positive samples in RSA and 85% in ELISA), the high serological titres, the positivity of all the tracheal samples collected during the production phase by MS PCR and the positivity by cultivation from tracheal swabs at the end-point (55 weeks after vaccination). In contrast, only one swab from a sternal *bursa* was positive in MS PCR, while all the joint and oviduct samples were negative. There was no evidence of vertical transmission. Different genotyping techniques were used to achieve a clear classification of the MS positive samples. The *vlhA* and the *obg* gene analysis showed that most of the strains were homologous with the vaccine, but some ambiguous samples were further investigated with the multi locus sequence typing (MLST) scheme which confirmed the homology.

**Conclusions:**

The development of a multi-technique approach to monitor vaccinated avian flocks, based both on serological and biomolecular methods, is advised as well as the use of effective genotyping techniques to analyse the MS strains circulating in high densely populated poultry areas.

**Electronic supplementary material:**

The online version of this article (10.1186/s12917-018-1669-8) contains supplementary material, which is available to authorized users.

## Background

*Mycoplasma synoviae* (MS) is a major pathogen responsible for economic losses in the poultry industry [1]. Although the infection occurs as a subclinical respiratory disease most of the time [1], MS can spread systemically to the joints and tendons in meat turkeys and broiler chickens; recently it has also been associated with eggshell apex abnormalities (EAA) in laying hens [2, 3]. In mixed infections with Newcastle disease and infectious bronchitis, MS can be found in the lower parts of the respiratory system [1].

MS infection can occur as a result of either horizontal or vertical transmission and its worldwide prevalence appears to be increasing [4], including Italy [5]. MS infection often leads to severe economic losses due to reduced body weight, poor feed conversion rate, high carcasses condemnation and increased antibiotic treatment; moreover in case of EAA it can involve increased egg fragility, drop in egg production and reduction of the average egg weight [3].

The most effective control strategies are focused on the development and maintenance of MS-free breeder flocks, but in case of infection the interruption of the vertical transmission represents an important goal for the practitioner. In order to contain or to arrest the vertical diffusion of MS several methods are available. Firstly, where MS prevalence is low, the culling of breeder flocks is considered the most effective approach [6], but it is rarely applied because of the high cost. However, the production of a MS-positive offspring could have even worse economic consequences than those arising from the culling of breeder groups.

Two more methods may be applied in order to reduce MS vertical transmission: the vaccination of breeders [7] or the application of a targeted antibiotic pulsatile treatment [Catania, personal communication]. These approaches represent useful tools for the producers to contain the risk of MS spread to the offspring and consequently to lower its prevalence in a geographical area.

Recently, a live vaccine (MS-H) developed in Australia (Vaxsafe MSH; Bioproperties Ltd., Ringwood, Victoria, Australia) has been licensed in Italy. The MS-H vaccine is a temperature-sensitive variant obtained by mutagenizing an Australian field isolate with N-methyl-N9-nitro-N-nitrosoguanidina [8]. The MS-H safety and the temperature sensitivity of the strain have been previously examined [9], while the possible development of biomolecular tests for its differentiation is still under investigation [10–12].

The availability of the vaccine in Italy and the producers’ need to better understand its behaviour in breeder flocks, as no previous report was available in this poultry sector, led to the development of a monitoring program of a vaccinated breeder group using samples collected for routine diagnostic purposes.

The aim of the present study was to evaluate the efficacy of the MS-H vaccine under field conditions in:

- a chicken breeder group measuring immunity response, MS-H presence, maintenance and tissue localization;
- embryos evaluating MS-H presence and maintenance;
- 1 day-old-chicks for serology.

In addition, several biomolecular techniques were applied to classify the MS positive samples and to confirm that the vaccine was the only strain circulating.

The development of a multi-technique scheme to distinguish the vaccine from the field strains circulating could be useful for *Mycoplasma* monitoring in breeders and especially in densely populated poultry areas (DPPAs).

## Results

### Breeders and MS-H vaccine

All the samples collected from the four barns 10 weeks after vaccination (14 weeks of age) were positive for MS in ELISA (40 positive samples out of 40), RSA (40 positive samples out of 40) and PCR (4 positive PCRs out of 4), confirming the correct application of the vaccination procedure. The ELISA titres were classified as positive in the 25% of the samples, as high positive in the 55% and as very high positive in the remaining 20% (Additional file 1).

### Recovery, viability and tissue distribution of MS-H vaccine in breeders

All the tracheal samples collected in each barn during the production phase (at 25, 30, 38, 44, 49, 54 weeks of age) were positive for MS by PCR (Additional file 2).

Post mortem examination of the 10 carcasses per barn did not show gross pathological lesions of MS. Nevertheless, pathological findings were reported from the sternal *bursa* of birds in barn 1, 5 and 2 (18 *sternal bursae*). The lesions ranged from mild inflammatory signs to fibrinous exudate. Moreover, at least one bird in each barn showed swollen joints (tibio-tarsal and/or interphalangeal joints), which were associated to increased synovial fluid or sero-catharral exudate. The swabs collected from sternal *bursae* and joints were submitted for specific MS PCR or for *Mycoplasma* cultivation.

All the tracheal swabs collected during post mortem examinations were positive by MS PCR (Additional file 3). Mycoplasma cultivation from tracheal swabs was also positive, showing typical egg-fried colonies on solid medium; moreover the 16S-rDNA-PCR-DGGE identified these positive samples by cultivation as *Mycoplasma synoviae*. Oviduct swabs were negative by both PCR and cultivation. All the specimens collected from the joints were also negative both in PCR and in cultivation, while the sternal *bursa* swab of a bird (in barn 2) out of the 3 collected was positive by MS PCR (Additional file 3).

### Assessment of vertical transmission of MS-H vaccine

A total of 150 embryonated eggs, collected at 30, 44, 49, 54 and 59 weeks, which correspond to the laying week 27, 41, 49, 51, 57, were negative for MS by isolation and specific PCR.

### Genotyping of MS positive samples in breeders

The 55 MS positive samples, resulting from MS PCR and *Mycoplasma* cultivation, were further investigated by molecular typing of their *vlh*A gene. Based on the *vlh*A analysis most of the samples were identical to the MS-H vaccine, as they were classified as type C, subtype 3 and group 13. However, one tracheal sample from barn 1 and one from barn 4 were classified as type E, group 11 (Table 1).

**Table 1:** Genotyping results of MS strains; for lepA, nanA, ruvB, ugpA and uvrA genes the allelic profile is presented. The Genebank Accession Numbers for each gene and strain are reported between brackets (Wks p.v. = weeks *post* vaccination)

Sample ID	Wks p.v.	Barn	vlhA	obg	lepA	nanA	ruvB	ugpA	uvrA
IZSVE/2017/860	MS-H	–	Type C3, group 13 (KY991388)	MS-H (KY991394)	2 (KY991412)	25 (KY991418)	19 (KY991430)	24 (KY991442)	21 (KY991454)
IZSVE/2013/2986–1	21	1	Type C3, group 13 (KY991382)	parental strain ts- (KY991400)	2 (KY991406)	25 (KY991424)	19 (KY991436)	24 (KY991448)	21 (KY991460)
IZSVE/2013/2986–4	21	4	Type C3, group 13 (KY991383)	reisolate ts- (KY991401)	2 (KY991407)	25 (KY991425)	19 (KY991437)	24 (KY991449)	21 (KY991461)
IZSVE/2013/4519–6	32	6	Type C3, group 13 (KY991384)	parental strain ts- (KY991402)	2 (KY991408)	25 (KY991426)	19 (KY991438)	24 (KY991450)	21 (KY991462)
IZSVE/2013/5404–6	40	6	Type C3, group 13 (KY991385)	MS-H (KY991403)	2 (KY991409)	25 (KY991427)	19 (KY991439)	24 (KY991451)	21 (KY991463)
IZSVE/2013/6965–6	50	6	Type C3, group 13 (KY991386)	parental strain ts- (KY991404)	2 (KY991410)	25 (KY991428)	19 (KY991440)	24 (KY991452)	21 (KY991464)
IZSVE/2014/368–2	55	2	Type C3, group 13 (KY991389)	MS-H (KY991395)	2 (KY991413)	25 (KY991419)	19 (KY991431)	24 (KY991443)	21 (KY991455)
IZSVE/2014/368–6	55	6	Type C3, group 13 (KY991390)	reisolate ts- (KY991396)	2 (KY991414)	25 (KY991420)	19 (KY991432)	24 (KY991444)	21 (KY991456)
IZSVE/2014/371–1	55	1	Type E, group 11 (KY991387)	reisolate ts- (KY991405)	2 (KY991411)	25 (KY991429)	19 (KY991441)	24 (KY991453)	21 (KY991465)
IZSVE/2014/373–12	55	3	Type C3, group 13 (KY991391)	parental strain ts- (KY991397)	2 (KY991415)	25 (KY991421)	19 (KY991433)	24 (KY991445)	21 (KY991457)
IZSVE/2014/374–12	55	4	Type E, group 11 (KY991393)	reisolate ts- (KY991399)	2 (KY991417)	25 (KY991423)	19 (KY991435)	24 (KY991447)	21 (KY991459)
IZSVE/2014/374–11	55	4	Type C3, group 13 (KY991392)	reisolate ts- (KY991398)	2 (KY991416)	25 (KY991422)	19 (KY991434)	24 (KY991446)	21 (KY991458)

Moreover, the *sp0B*-associated GTP binding gene (*obg*) of all the positive samples were sequenced and analysed in three nucleotide positions (pos. 178, 367 and 629). Briefly, the analysis of the point mutation in position 629 (C > T) reveal the transition from a C in the MS-H ts + strain to a T in a re-isolated ts- strain, which was detected for the first time at week 25 in barn 4. Moreover, reisolated ts- strains were detected in barn 1, 4, 5 and 6 at 59 weeks.

Based on the analysis of the *obg* gene [18, 19] in position 367 (A > G), 4 samples (at week 25 in barn 1, then at week 36 in barn 6, at week 50 in barn 6 and finally at week 59 in barn 3) were classified as parental strain ts- (a vaccine parent strain, non thermosusceptible). The *vlh*A gene of these 4 samples was previously defined as type C3, group 13 (Table 1).

In order to detect the presence of wild strains, 6 samples not clearly classified by previous methods were further investigated by MLST. Their MLST allelic profile (MLST ST) was identical to the MS-H vaccine, confirming that there was no other MS strain circulating during the field study (Table 1).

### MS immunity in breeders and offspring

Just over 80% (666 of 840) of all tested samples were positive by the RSA. However, there were differences in the percentage of positive samples over time: week 25 (38%), 30 (80%), 38 (100%), 44 (62%), 49 (75%), 54 (100%) and 60 (100%). The ELISA results showed an evident seroconversion in the all tested samples (717 positive sample out of 840–85%), with modifications during the time week 25 (75%), 30 (100%), 38 (100%), 44 (83%), 49 (83%), 54 (83%) and 60 (85%) and between flocks (Table 2). The positive titres showed a wide range of distribution: most of the samples (65%) were classified as class A (positive), whereas the percentage of class B (high positive) was 32% and only 3% were included in the C class (very high positive). Four barns (2, 4, 5, and 6) showed very high positive titres and only barn 6 reached the 65% of positivity (C class). The mean titres are reported in Table 2.

**Table 2:** ELISA serological titres of breeder flocks represented in Number of Positive Samples (NPS) and percentage (%), the mean titre is reported above

	Weeks of age	25th week	30th week	38th week	44th week	49th week	54th week	59th week	
Barn									Total
1	NPS (%)	6 (30%)	20 (100%)	15 (75%)	16 (80%)	10 (50%)	10 (50%)	10 (50%)	87 (62%)
1	Mean titre	1653	5152	4430	2305	3599	4105	3577	4003
2	NPS (%)	18 (90%)	20 (100%)	10 (50%)	16 (80%)	10 (50%)	14 (70%)	16 (80%)	104 (74%)
2	Mean titre	1953	2886	2422	3948	2538	1783	2310	2633
3	NPS (%)	16 (80%)	20 (100%)	20 (100%)	20 (100%)	20 (100%)	18 (90%)	20 (100%)	134 (96%)
3	Mean titre	3938	4607	5853	2210	2837	2482	3271	3557
4	NPS (%)	20 (100%)	20 (100%)	20 (100%)	20 (100%)	20 (100%)	20 (100%)	18 (90%)	138 (99%)
4	Mean titre	3278	4616	3886	5236	2769	5314	2874	4105
5	NPS (%)	20 (20%)	20 (100%)	20 (100%)	12 (60%)	20 (100%)	20 (100%)	20 (100%)	132 (94%)
5	Mean titre	2739	3365	3561	2348	3391	4633	5802	3733
6	NPS (%)	10 (50%)	20 (100%)	20 (100%)	16 (80%)	20 (100%)	18 (90%)	18 (90%)	122 (87%)
6	Mean titre	14,583	4536	3668	2261	3327	6639	2473	4795
	Total	90 (75%)	120 (100%)	105 (100%)	100 (83%)	100 (83%)	100 (83%)	102 (85%)	717 (85%)
	Mean titre	4158	4193	4096	2849	3079	4291	3436	3753

About the 57% of sera from 1-day-old chicks was positive at the 3 sampling times (Table 3); all the positive samples showed titres below 4.000 (positive).

**Table 3:** Results of 1-day-old chicken *sera* tested in MS ELISA (chicks hatched from vaccinated birds), expressed as number of positive and negative samples and in percentage between brackets

Week of layed eggs	Samples number		
	Negative	Positive	Total
41	38 (63%)	22 (37%)	60
46	30 (75%)	10 (25%)	40
51	- (0%)	60 (100%)	60
Total	68 (43%)	92 (57%)	160

## Discussion

The increasing prevalence of MS in many countries shows that this pathogen represents a persistent challenge for the poultry industry. The vertical transmission of MS can be considered a critical point to control its spread to the offspring. The control programs previously applied to the poultry industry have enabled the eradication of important *Mycoplasma* species, such as MG where a high reduction of prevalence has been achieved at least in the European countries, while sporadic outbreaks of *Mycoplasma meleagridis* and *Mycoplasma iowae* are occasionally reported in backyard flocks [14] and industrial turkeys respectively [24, 25]. The eradication measures commonly applied for *Mycoplasma* species seem to have been ineffective for MS which could be related to the lower attention paid up to now to this bacterial species. The implementation of biosecurity measures, the targeted antibiotic pulsatile treatment of breeders or the vaccination could be applied to reduce the spread of MS-positive offspring, without resorting to the cull of breeders.

The aim of this study was to analyse the MS vaccine behaviour (MS-H) in a broiler breeder farm with a high biosecurity level. In particular, it was directed to evaluate the laboratory tests available to screen the animals and to define correctly the sanitary status of breeder groups, which could also be useful for the authorization in intra-community exchanges (UE) of embryonated eggs or 1-day-old chicks (2009/158/CE).

The thermo-susceptible MS-H strain showed a wide diffusion in the breeder group confirmed by the positivity in PCR during the entire production period, in addition the wide spread of the strain, is confirmed by the serological data reported in this study, in which most of the tested population showed a strong seroconversion. It is interesting to highlight that while the MG vaccine (TS-11®) is produced with a similar technology to the MS-H, their serological behaviour looks different. Flocks vaccinated with TS-11® have a lower seroconversion rate of around 60% of birds as well as lower mean titres as compared to MS-H vaccinated flocks. As reported by Barbour et al. (2000) [26], the ELISA OD rate at the peak (23 weeks after vaccination with ts-11) was 46.6% in flock 1 and the TS-11® is not commonly recovered after the 30th week after vaccination. Actually, in the present study, the serum samples of birds in many barns (3, 4, 5, and 6) showed a100% positivity for MS with ELISA at different sampling times and the rate of positivity was particularly high in the last 30 weeks of production, while the MS-H vaccine was administered at 4 weeks of age. The positive titres were very high demonstrating a strong immune reaction against the vaccine.

This strong serological behaviour could be explained by the high MS-H persistence in the population and by its possible recirculation in the host, which provides a persistent immunogenic stimulus. This high seroconversion in breeders is then transferred to 1-day-old chicks, as MS antibodies were clearly detected in the offspring; but these findings should be confirmed by further studies on a larger number of samples. These preliminary results could represent a disadvantage for the trade in 1-day-old chicks, until a DIVA serological test is developed.

In addition, the vaccine strain was also highly persistent during the period of the study as it was still being isolated from the trachea of birds at the end of the cycle (55th weeks after vaccination). This strain seems to be able to recirculate inside the population and to maintain itself in the flock. This behaviour is similar to that expressed by wild strains, which are associated with positivity by PCR during the entire life of the breeders. It is evident that the PCR screening of tracheal swabs is not enough to discriminate a vaccinated group from an infected one and more discriminatory tests are required.

The long term persistence of the vaccine is confirmed by the culture method of MS from tracheal samples at the end of the cycle. The isolation of mycoplasma by cultural method demonstrates the viability of the strain and its presence in high quantity. The vaccine was isolated at the end of the cycle from the upper respiratory tract, whereas it is important to highlight that there was no evidence of MS in other target sites, such as oviduct and joint, and that one swab taken from an abnormal sternal *bursa* was positive in the MS PCR. The detection of MS DNA in the sternal bursa of one bird could be probably due to its spread within the body, but further studies are needed to confirm this data.

As only tracheal samples were positive by cultivation, the vaccine strain, unlike the wild types, seemed unable to colonize different tissues other than the upper respiratory tract.

These results confirm, as reported for the ts-11 vaccine [27], that the MS-H seemed incapable of spreading and colonizing the oviduct, which was also shown by the negative results of the oviducts and, therefore, of the embryonated eggs. The laboratory findings of the present study suggest that there was no vertical spread of the MS vaccine, but further large scale studies should be carried out in view of the recent reports of MG vaccine transmission in the USA [28].

The *vlhA* gene sequencing was applied as it is a helpful tool to classify different isolates and in some specific cases or geographic areas it could also be used to discriminate the vaccine from wild strains [21, 22]. Recently, the *vlhA* technique was not always found to be able to discriminate the vaccine in Italy [20], as a wild strain isolated in this geographical area showed complete homology to the MS-H in the *vlhA* gene sequence. An additional biomolecular test, based on the *obg* gene analysis [19, 29, 20] was introduced to classify all the positive samples as MS-H strain, MS-H reisolates (ts + and ts-) or parental strains ts-.

The analysis of the *obg* gene sequence defined most of the samples as MS-H, but it is important to highlight that some samples were classified as re-isolates ts-, confirming the possible lack of thermosusceptibility as previously reported by Noormohammadi et al. (2003) [30]. In the present study the single point mutation associated with the lack of thermosusceptibility was detected as early as week *post* vaccination (p.v.). 21 in barn 4 and then at 55 weeks p.v. in barn 1, 4, 5 and 6. It is interesting to report that the mutation in position 629 was not confirmed in each barn at every sampling time; for example in barn 4 the point mutation was not recovered until the end of the trial. These results suggest that the 2 vaccine types (MS-H ts + and reisolate ts-) could be present together in the population. It is important to report that from the 25th week (21 weeks after vaccination), at many sampling times, at least one ts- reisolate was detected in the six barns. Four MS positive samples, defined as type C3 group 13 for the *vlhA* gene, were classified as parental strain ts- based on the *obg* gene analysis.

Moreover, the analysis of 5 housekeeping genes basing on the MLST scheme [23] was applied in order to exclude an improper classification and the presence of any other strain circulating. The results confirmed the complete homology of all the samples, including the ambiguous ones, to the MS-H vaccine in all the 5 genes.

Based on the *vlhA* genotyping techniques [16, 17], all the strains were classified as type C, subtype 3 and group 13, which is the same genotype as the vaccine, with the exception of 2 samples (barn 1 and 3), which showed a different *vlhA* sequence classified as type E and group 11.

These data highlight the possible rearrangement of the *vlhA* gene, which is considered a common feature of *Mycoplasma* species [30, 31]. They also show that the MS-H vaccine was maintained in the avian group as a main population with the same *vlhA* sequence (type C3, group 13) and a minor population with a different *vlhA* type (type E, group 11). Moreover, it is important to emphasise that this mutated population was detected only in 2 samples by isolation and PCR and that the different *vlhA* type (type E, group 11) is the same previously reported by Bayatzdeh et al. (2014) [22] in a similar field trial. While few reports exist, studies have shown a change of the *vlhA* sequence in vaccinated birds and in both cases the same kind of genetic change was seen; thus it is possible to speculate that MS possesses a specific and organized ability to rearrange genetic segments the *vlhA* gene [30]. Of course further studies are necessary to substantiate this hypothesis.

## Conclusions

The MS-H vaccine showed a wide diffusion and a great persistence in the groups, evidenced by a clear and high seroconversion of the vaccinated chickens and their progeny comparable to the behaviour of the wild strain. The *vlhA* method alone seems not always effective in discriminating the vaccine from wild strains, as recently reported by Catania et al. (2016) [20]; in fact two samples were classified as type E – group 11, instead of type C3 - group 13. In a similar way, the *obg* gene analysis was not always successful in the definition of the vaccine or wild strain, as the four samples classified as parental strain ts- showed complete homology to the MS-H vaccine ST, based on the MLST scheme.

Moreover, no clinical or pathological signs characteristics of MS infection were found during the whole production cycle. Consequently, based on these results, the use of the *obg* gene for the MS-H vaccine seems insufficiently discriminatory and up to now the application of different biomolecular techniques together could represent an effective laboratory system to monitor vaccinated populations.

The development and validation of biomolecular and serological DIVA methods, as well as genotyping studies on the MS population in DPPAs, are strongly desirable to improve monitoring laboratory activities on avian flocks.

## Methods

### Site description and history

A broiler chicken breeder farm, with high biosecurity measures, in a DPPA in Italy was the target of the present study. The farm had been monitored for *Mycoplasma gallisepticum* (MG) and *Mycoplasma synoviae* (MS) for several years before the study started and was shown to be MG-*free*, whereas there had been periodic outbreaks of MS.

The samples (sera, tracheal swabs and carcasses) were sent to the Avian Medicine Laboratory of the Istituto Zooprofilattico Sperimentale delle Venezie (IZSVe, Legnaro – Padova, Italy) for diagnostic and monitoring purposes and, in agreement with the farmer, were used for further investigations of MS.

### Breeders and MS-H vaccine

Approximately 28,000 one-day-old chicks (mixed-sex) were bought as MG- and MS-free. They were allocated to separate farm and into 4 different barns: 3 female barns (2 b with chicks of Ross genetic line and 1 b with chicks of Cobb genetic line) and 1 male barn in 2-floor pens with mixed genetics, until transfer to the production site, which took place at 23rd week of age.

Before MS-H vaccination, birds were monitored serologically and by PCR to exclude the presence of MS infection. 30 MS ELISA (Idexx® USA), 30 rapid serum agglutination (RSA) tests (IDVET, Montpellier) [6] and 30 MS PCRs [13] on tracheal swabs were performed on randomly selected birds in each barn. At 4 weeks of age all the birds were vaccinated for MS with MS-H vaccine (Vaxsafe MSH; Bioproerties Ltd., Ringwood, Victoria, Australia) following the manufacturer’s instructions.

Ten weeks after the MS-H vaccination (14 weeks of age), 10 randomly selected birds in each barn were monitored serologically and by PCR comprising each barn 10 MS ELISA, 10 MS RSA and 10 tracheal swabs for MS PCR, for a total amount of 40 MS ELISA, 40 MS RSA and 4 MS PCR.

**Fig. 1:**
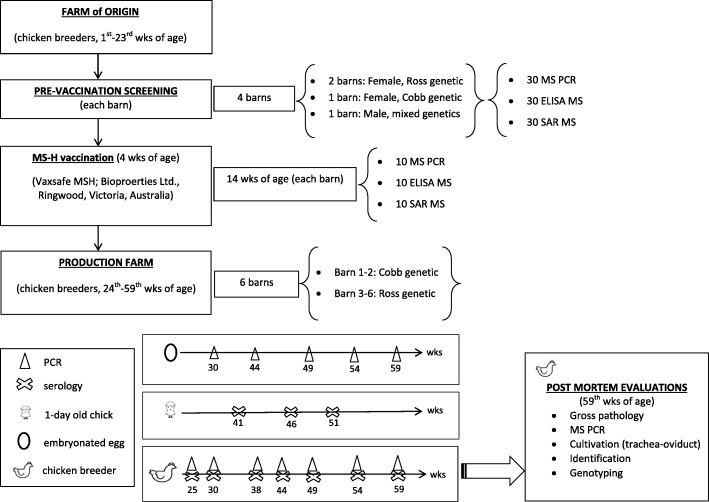
Flow chart of the animal housing and the protocols applied during the study

The production farm (from weeks 23rd to 59th) consisted of 6 b of broiler breeders: barn 1 and 2 housed birds of the Cobb genetic, whereas barns 3, 4, 5 and 6 held Ross 308 chicks. The organization of the animal housing and the protocols applied are shown in Fig. 1.

### Recovery, viability and tissue distribution of MS-H vaccine in breeders

At the 23rd week of age, animals were moved to the production farm and divided in 6 groups as previously described.

Animals of each barn were monitored at fixed times: 25, 30, 38, 44, 49, 54 and 59 weeks of age. At every time interval, 10 tracheal swabs/barn were sent to the IZSVe for specific MS PCR [13].

At 59 weeks of age, basing on the farmer’s standards for animals kept commercially, the production period was considered finished and all the animals were sent to the abattoir, where a properly approved stunning and killing method was applied in respect of the nationally implemented EU legislation.

Then, for each barn ten animal carcasses were sent to the IZSVe for complete post mortem examination, particularly directed to the detection of MS. For each barn, 10 tracheal and 10 oviduct swabs were collected during necropsy for specific MS PCR and cultivation. Additional samples were collected when gross pathological lesions characteristic of MS were found in target organs (i.e. air sacs, tibio-tarsal joints, sternal bursa). Consequently, 3 abnormal sternal bursa and 11 joints were tested either by PCR or *Mycoplasma spp.* cultivation.

DNA was extracted from the collected specimens using QIAmp® DNA mini kit (Qiagen®) and tested by a specific MS PCR [13].

Furthermore, swabs were submitted for *Mycoplasma spp.* isolation in Mycoplasma Experience (ME) broth medium (Reigate, UK) following the internal laboratory procedure and incubated at 37 °C under CO_2_ conditions for at least 15 days. Briefly, during this time the cultures were checked daily and when a change in colour or turbidity was seen, the broth was inoculated onto an agar plate (ME, Reigate, UK) to evidence the presence of typical egg-fried colonies [14]. The inoculated agar plates were checked for 15 days and considered negative if no sign of growth was seen after this period of time [14]. DNA was extracted from broths of suspect samples using the High Pure PCR Template Preparation Kit (Roche®) and *Mycoplasma* species identification was performed with the 16S-rDNA-PCR-DGGE (denaturing gradient gel electrophoresis) [15].

### Assessment of vertical transmission of MS-H vaccine

When broiler breeders were 30, 44, 49, 54, 59 weeks of age 30 embryonated eggs were collected and tested for MS by isolation and specific MS PCR. The specimens collected from the embryos were swabs of the internal surface of the yolk sac and the trachea, which were analysed in pool of 10 samples for *Mycoplasma* spp. cultivation and specific MS PCR, as previously described.

### Genotyping of MS positive samples in breeders

As the present study was carried out in a commercial farm, two main genotyping methods were applied to all the MS positive samples to confirm that the vaccine was the only strain circulating. These samples were analysed in the variable lipoprotein hemagglutinin A (*vlhA*) gene [16, 17] and the *sp*0B-associated GTP binding globulin gene (*obg*) [18, 19]. Where the *vlhA* and *obg* gene analysis led to ambiguous results, samples were further investigated by multi locus sequence typing scheme (MLST) of 5 specific housekeeping genes [23]. The oligonucleotide primers used in this study and the amplification product size are reported in Table 4. The positive PCR products were purified using the A’SAP PCR clean up kit following the manufacturer’s instructions and sequenced in both directions using the dye terminator sequencing method. The sequences were analysed using Molecular Evolutionary Genetics Analysis6 (MEGA6) software.

**Table 4:** Gene targets and primer details

Gene target	Primer forward	Primer reverse	PCR amplicon size (bp)
vlhA	5′-ATT AGC AGC TAG TGC AGT GGC C-3’	5′-AGT AAC CGA TCC GCT TAA TGC-3’	315–372
obg	5′-GTT GAT AAA GGT GGA CCA G − 3′	5′-TTA GTG CAG ATA TCT CAA TG-3’	841
nanA	5’-TTGAAAAACAAAAAGTTGATGGAA-3′	5’-ATGATTGCATTAGCGCCTTT-3’	544–559
ugpA	5’-TTAGATTTGCAATCGGTTTTAGA-3′	5’-CCGCGTCAGTTGGACTATTT-3’	537
ruvB	5’-GAATGCCTGGAATGGGTAAA-3’	5’-TGTCAATGATTCCAGCGTTT-3’	546
lepA	5’-AGCGCCGATGTTGAYAGAGT-3’	5’-GAGTGCTTCTTTGGCTGGAT-3’	462
uvrA	5’-CKGCAAACATGGTTTCTTCG-3’	5’-TGGGTTCTTTTACATGCGGTA-3’	468

### MS immunity in breeders and offspring

At fixed times (25, 30, 38, 44, 49, 54 and 59 weeks of age) 20 blood samples from each barn were collected and analysed at the IZSVe for specific MS serology. Blood samples were tested for MS ELISA and Rapid Serum Agglutination (RSA) as above. Concerning the MS ELISA, positive titres were classified as A: positive titre of < 4.000 OD (low positive), B: positive titre < 10.000 OD (high positive) and C positive titre > 10.000 OD (very high positive). Since the monitoring programs are not commonly applied to 1-day-old chicks, a small number of serum samples (160 *sera* from chicks hatched from vaccinated birds) were randomly collected from several barns and tested in MS ELISA. The time of collection was 41, 46 and 51 weeks of age of the breeders.

## Additional files

Additional file 1: ELISA serological titres of chickens 10 weeks after vaccination. Results are represented in Number of Positive Samples (NPS) and percentage (%), the mean titre is reported above. (DOCX 13 kb)

Additional file 2: Results of the MS PCR from tracheal swabs per barn during the production phase (+ = positive result; − = negative result). All the samples tested during the time resulted positive at each sampling time. (DOCX 14 kb)

Additional file 3: Results of the MS PCR from trachea, oviduct, joint and sternal bursa collected during post-mortem activities (+ = positive result; − = negative result; n.d. = not done). All the tracheal swabs resulted positive for MS in PCR, whereas oviduct and joint swabs resulted negative. (DOCX 13 kb)

## Abbreviations

DGGE: Denaturing Gradient Gel Electrophoresis
DPPAs: Densely Populated Poultry Areas
EAA: Eggshell Apex Abnormalities
IB: Infectious Bronchitis
IZSVe: Istituto Zooprofilattico Sperimentale delle Venezie
ME: Mycoplasma Experience
MEGA: Molecular Evolutionary Genetics Analysis
MG: Mycoplasma gallisepticum
MLST: Multi Locus Sequence Typing
MS: Mycoplasma synoviae
MS-H: Mycoplasma synoviae vaccine
ND: Newcastle Disease
obg: sp0B-associated GTP binding globulin gene
OD: Optical Density
RSA: Rapid Serum Agglutination test
vlhA: variable lipoprotein haemagglutinin A
