# Supplementary material for: Application of different laboratory techniques to monitor the behaviour of a Mycoplasma synoviae vaccine (MS-H) in broiler breeders
Source: BMC Vet Res. 2018 Nov 20;14:357. doi: 10.1186/s12917-018-1669-8 (PMC6245925; doi:10.1186/s12917-018-1669-8)
Supplement: Supplementary file 1 — ELISA serological titres of chickens 10 weeks after vaccination. Results are represented in Number of Positive Samples (NPS) and percentage (%), the mean titre is reported above. (DOCX 13 kb) [file 12917_2018_1669_MOESM1_ESM.docx]

Additional file 1. ELISA serological titres of chickens 10 weeks after vaccination. Results are represented in Number of Positive Samples (NPS) and percentage (%), the mean titre is reported above.

| **Barn** | **ELISA results** | |
| --- | --- | --- |
| **1** | NPS (%) | (10)  (100%) |
| **1** | Mean titre | 5719 |
| **2** | NPS (%) | (10)  (100%) |
| **2** | Mean titre | 6714 |
| **3** | NPS (%) | (10)  (100%) |
| **3** | Mean titre | 8463 |
| **4** | NPS (%) | (10)  (100%) |
| **4** | Mean titre | 9172 |
