# Supplementary material for: Application of different laboratory techniques to monitor the behaviour of a Mycoplasma synoviae vaccine (MS-H) in broiler breeders
Source: BMC Vet Res. 2018 Nov 20;14:357. doi: 10.1186/s12917-018-1669-8 (PMC6245925; doi:10.1186/s12917-018-1669-8)
Supplement: Supplementary file 2 — Results of the MS PCR from tracheal swabs per barn during the production phase (+ = positive result; − = negative result). All the samples tested during the time resulted positive at each sampling time. (DOCX 14 kb) [file 12917_2018_1669_MOESM2_ESM.docx]

Additional file 2. Results of the MS PCR from tracheal swabs per barn during the production phase (+ = positive result; - = negative result).

|  | WEEK OF AGE | | | | | |
| --- | --- | --- | --- | --- | --- | --- |
| BARN N° | 25 TH | 30 TH | 38 TH | 44 TH | 49 TH | 54 TH |
| 1 | + | + | + | + | + | + |
| 2 | + | + | + | + | + | + |
| 3 | + | + | + | + | + | + |
| 4 | + | + | + | + | + | + |
| 5 | + | + | + | + | + | + |
| 6 | + | + | + | + | + | + |
