# Supplementary material for: Application of different laboratory techniques to monitor the behaviour of a Mycoplasma synoviae vaccine (MS-H) in broiler breeders
Source: BMC Vet Res. 2018 Nov 20;14:357. doi: 10.1186/s12917-018-1669-8 (PMC6245925; doi:10.1186/s12917-018-1669-8)
Supplement: Supplementary file 3 — Results of the MS PCR from trachea, oviduct, joint and sternal bursa collected during post-mortem activities (+ = positive result; − = negative result; n.d. = not done). All the tracheal swabs resulted positive for MS in PCR, whereas oviduct and joint swabs resulted negative. (DOCX 13 kb) [file 12917_2018_1669_MOESM3_ESM.docx]

Additional file 3. Results of the MS PCR from trachea, oviduct, joint and sternal bursa collected during post-mortem activities (+ = positive result; - = negative result; n.d. = not done).

|  | 59 WEEKS OF AGE | | | |
| --- | --- | --- | --- | --- |
| BARN N° | TRACHEA | OVIDUCT | JOINTS | STERNAL BURSA |
| 1 | + | - | - | - |
| 2 | + | - | - | + |
| 3 | + | - | - | n.d. |
| 4 | + | - | - | n.d. |
| 5 | + | - | - | - |
| 6 | + | - | - | n.d. |
